# T218. IDENTIFICATION OF FIRST-EPISODE PSYCHOSIS SUBGROUPS BASED ON POSITIVE SYMPTOM DOMAINS AND THEIR SOCIODEMOGRAPHIC AND CLINICAL CORRELATES

**DOI:** 10.1093/schbul/sby016.494

**Published:** 2018-04-01

**Authors:** Chiara Galletti, Luca Pauselli, Marc Manseau, Alfonso Tortorella, Michael T Compton

**Affiliations:** 1 University of Perugia; 2 Columbia University; 3 New York University

## Abstract

**Background:**

Although the heterogeneous nature of psychosis is well established in the literature, genetics, neurophysiology, and neuroimaging have not yet succeeded in an unequivocal classification of the diverse clinical presentations. For the time being, the field relies mostly on a symptom-based, descriptive diagnostic system. The purpose of the current study was to use seven previously identified positive symptom domains to identify possible subgroups of first-episode psychosis (FEP), using cluster analysis. In addition, we tested whether possible subgroups differed across a number of sociodemographic and clinical variables.

**Methods:**

We analyzed data from a large FEP sample (n=247) to identify possible clinical subgroups of psychosis based on positive symptoms; specifically, delusion and hallucination domains resulting from previous factor analyses of Scale for the Assessment of Positive Symptoms (SAPS) items in this sample. A rigorous methodology was applied to perform cluster analysis and identify FEP subgroups. Kruskal-Wallis tests with pairwise post-hoc comparisons were used to check the differences in each positive symptom domain between the subgroups. Bivariate analyses comparing subgroups on a number of sociodemographic and clinical characteristics were performed.

**Results:**

Five FEP subgroups were identified based on the severity of seven domains of delusions and hallucinations. Three of them (Mild Positive Symptoms Subgroup, Moderate Positive Symptoms with Sin/Guilt and Jealousy Delusions Subgroup, Severe Positive Symptoms Subgroup) shared a similar psychopathological profile, with typical psychotic symptoms differing in severity. Another subgroup was characterized by high severity on typical and atypical symptoms (Severe Mixed Atypical Positive Symptoms Subgroup), and the other by high severity in somatic delusions (Somatic Delusions Subgroup). The five subgroups did not significantly differ in terms of gender, age at onset, family history, mode of onset of psychosis, premorbid functioning, and alcohol and non-cannabis drug use disorders, though some potential signals were identified, (but not reaching statistical significance due to small sample sizes in the subgroups). Significantly different prevalence rates of cannabis use disorder were found across the five subgroups.

**Discussion:**

In our analysis, five possible FEP subgroups were identified based on the severity of seven domains of delusions and hallucinations: three of them shared a similar psychopathological profile differing in severity, and two were characterized by different atypical symptoms. Despite several acknowledged limitations, our results highlight the potential to identify clinical phenotypic subgroups of FEP, which may be helpful in future research aimed at filling the gaps between clinical, neuropathological, and genetic explanations of psychosis etiology. Such an approach may also lead to better targeted preventive interventions and more individualized and effective treatments.

